# Does a Consumer-Targeted Deprescribing Intervention Compromise Patient-Healthcare Provider Trust? †

**DOI:** 10.3390/pharmacy6020031

**Published:** 2018-04-16

**Authors:** Yi Zhi Zhang, Justin P. Turner, Philippe Martin, Cara Tannenbaum

**Affiliations:** 1 Faculté de Pharmacie, Université de Montréal, Montreal, QC H3T 1J4, Canada; yi.zhi.zhang@umontreal.ca (Y.Z.Z.); philippe.martin@umontreal.ca (P.M.); cara.tannenbaum@umontreal.ca (C.T.); 2 Centre de Recherche, Institut Universitaire de Gériatrie de Montréal, Montréal, QC H3W 1W5, Canada

**Keywords:** deprescribing, trust, Doctor-Patient Relations, potentially inappropriate medications, aged, aged 80 and over

## Abstract

One in four community-dwelling older adults is prescribed an inappropriate medication. Educational interventions aimed at patients to reduce inappropriate medications may cause patients to question their prescriber’s judgment. The objective of this study was to determine whether a patient-focused deprescribing intervention compromised trust between older adults and their healthcare providers. An educational brochure was distributed to community-dwelling older adults by community pharmacists in order to trigger deprescribing conversations. At baseline and 6-months post-intervention, participants completed the Primary Care Assessment Survey, which measures patient trust in doctors and pharmacists. Changes in trust were ascertained post-intervention. Proportions with 95% confidence intervals (CI), and logistic regression were used to determine a shift in trust and associated predictors. 352 participants responded to the questionnaire at both time points. The majority of participants had no change or gained trust in their doctors for items related to the choice of medical care (78.5%, 95% CI = 74.2–82.8), communication transparency (75.4%, 95% CI = 70.7–79.8), and overall trust (81.9%, 95% CI = 77.9–86.0). Similar results were obtained for participants’ perceptions of their pharmacists, with trust remaining intact for items related to the choice of medical care (79.4%, 95% CI = 75.3–83.9), transparency in communicating (82.0%, 95% CI = 78.0–86.1), and overall trust (81.6%, 95% CI = 77.5–85.7). Neither age, sex nor the medication class targeted for deprescribing was associated with a loss of trust. Overall, the results indicate that patient-focused deprescribing interventions do not shift patients’ trust in their healthcare providers in a negative direction.

## 1. Introduction

With increasing age comes an accumulation of comorbidities [1,2], medications [3], and potentially inappropriate prescriptions [4]. A medication is deemed potentially inappropriate when the potential for harm outweighs the potential for benefit, and when safer alternatives exist [5,6]. Use of potentially inappropriate medications such as benzodiazepines has been associated with a higher risk of falls, fractures, cognitive impairment and mortality in older adults [7,8,9,10,11,12].

Patient education about medication harms is a novel method to drive conversations around deprescribing inappropriate medications. A direct-to-consumer educational intervention was successful in reducing inappropriate benzodiazepine use in community-dwelling older adults by 27% over a six month time period [13]. Almost two-thirds of recipients initiated a conversation about deprescribing with their healthcare provider after receipt of the intervention. Healthcare providers were generally supportive of patients’ requests to taper their sedative-hypnotics. However, one reason stated by some participants for not opting to discuss discontinuation was trust in their healthcare provider’s decision to prescribe the medication in the first place [14]. “When my doctor prescribes something for me, I know it’s not junk, I know it’s good for me. And I don’t question it,” said a 72-year-old man who was interviewed after the study [14]. 

The quandary of asking patients to confront prescribers about potentially inappropriate prescriptions raises issues of patient-prescriber trust and the possibility that patients may start to mistrust their prescriber’s judgment. Trust has been defined as the characteristic that gives meaning to the relationship between the patient and their healthcare provider [15]. High levels of patient trust have been linked to positive outcomes, such as increased adherence to treatment recommendations including medication use [16]. Trust also led 71% of community-dwelling older adults who were surveyed to report a willingness to deprescribe a medication if their doctor recommends it [17]. The healthcare professional’s attitude can therefore have a significant influence on a patient’s medication taking behavior and can act as an enabler or a barrier to deprescribing [18,19].

## 2. Aim

The aim of this study was to determine whether a patient-focused educational intervention to reduce inappropriate medications compromises trust between community-dwelling older adults and their healthcare providers.

## 3. Materials and Methods

### 3.1. Population

Community-dwelling older men and women (aged ≥65 years) in Montreal, Canada, were invited to participate in a study focused on safe medication management [20]. Inclusion criteria were chronic use (≥3 months) of potentially inappropriate medications: benzodiazepines, long-acting sulfonylureas, first-generation antihistamines, and non-steroidal anti-inflammatory drugs. These medication classes were selected from the 2012 Updated Beers Criteria list of medications to avoid in the elderly, based on their high frequency of use among Canadian older adults [20,21]. Full details of the study methodology have been described previously [20]. Briefly, participants were screened for eligibility and contacted by their pharmacist and invited to participate in the study. A research assistant solicited the participant’s informed consent and contacted participants at several time points, including baseline and 6-months, to query participants’ perceptions parallel to the educational intervention. The patient educational intervention included an 8-page brochure on the harms associated with their medication use, and suggestions for substitution with safer drug or non-drug therapy. As part of the trial protocol, the pharmacist was asked to contact the patient’s family doctor by faxing an evidence based tool called a pharmaceutical opinion, as per professional standards in Quebec. The pharmaceutical opinion was based on a validated prototype and contained information including personalized patient information, evidence-based information on medication harms, deprescribing and recommended alternatives [22].

### 3.2. Measurements

At baseline a research assistant administered a survey to participants querying age, sex, medication class, level of trust in their doctor, and level of trust in their pharmacist. At one week, after receipt of the intervention, participants were asked about their intent to discuss discontinuation of their medication with a healthcare provider. The three questions about trust were taken from the validated *Primary Care Assessment Survey* (PCAS) developed by Safran et al. used to measure domains of healthcare [23]. All eleven scales within the PCAS have excellent measurement properties with trust having good internal consistency and reliability (Cronbach’s alpha 0.86) [23]. Participants rated their agreement with statements regarding their doctor and pharmacist’s choice of medical care, transparency in communicating, and overall trust. Responses were rated on a 5-point Likert-type scale for level of trust in both healthcare providers separately (Table 1). The three questions about trust in their doctor and the three questions about trust in their pharmacist were re-administered at 6-month follow up.

### 3.3. Outcome

The primary outcome was a change in participants’ level of trust in their doctor or pharmacist on any of the three trust items between baseline and 6-months after receiving the educational deprescribing intervention.

### 3.4. Statistical Analysis

Proportions and 95% confidence intervals were used to describe endorsement of the response options to each question. To measure change in trust, a minimum of a single unit of change on the 5-point Likert-scale between baseline and 6-months was considered a change in level of trust. To facilitate analysis, Likert-scale response options were collapsed to indicate trust (strongly agree or agree with the first statement, strongly disagree or disagree with the second statement, and an answer of mostly or completely with the third statement). Participants who had an increase in trust or no change from baseline were combined to create the dichotomous outcome of non-compromised trust. Participants with non-compromised (or intact) trust were compared to those with a loss of trust, or compromised trust. Logistic regression was used to determine whether age, sex, medication class or intent to discuss tapering off their medication were associated with a loss of trust. All data were analyzed using the Statistical Package for the Social Sciences, version 24 (SPSS, Chicago, IL, USA).

### 3.5. Ethics

The study protocol was approved by the Research Ethics Board of the Centre de Recherche de l’Institut de Gériatrie de Montréal, Canada on 17 September 2013.

## 4. Results

Results from the first 352 participants enrolled in the trial were analyzed for this study as part of a preliminary analysis of the D-PRESCRIBE trial. Three responses to the doctor portion of the survey were incomplete, along with five responses to the pharmacist portion. Table 2 reports participant demographics. Mean age was 74.3 (standard deviation 6.4 years, range 66–92), and the majority of participants were women (64.8%). Of the 352 participants, 58.2% were taking benzodiazepines, 25.5% long-acting sulfonylureas, 13.2% non-steroidal anti-inflammatory drugs, and 3.2% first-generation antihistamines. At one week post-intervention, 62.5% of participants intended to speak to a healthcare provider about making changes to their medication, while 37.5% had no intention of doing so.

Table 3 presents baseline and 6-month follow up data related to participant trust in their healthcare provider. At baseline, most participants agreed or strongly agreed about trusting their doctor’s judgment about their medical care. The majority disagreed or strongly disagreed that their doctor would hide a treatment mistake from them. In terms of overall trust, 61.9% of participants expressed complete trust in their doctor. For the pharmacist, most participants agreed or strongly agreed about trusting their pharmacist’s judgment about their medical care. The majority disagreed or strongly disagreed that their pharmacist would hide a treatment mistake from them. In terms of overall trust, 48.3% of participants completely trusted their pharmacist.

Table 4 shows the extent to which participants’ perceptions of trust in their doctor or pharmacist changed as a result of receiving the deprescribing intervention. Between baseline and 6-month follow-up, most participants had no change or increased trust in their doctor for questions pertaining to the choice of medical care (78.5%), communication transparency (75.4%), and overall trust (81.9%). Similarly, most participants had no change, or increased trust in their pharmacist for questions pertaining to the choice of medical care (79.4%), communication transparency (82.0%), and overall trust (81.6%). 

**Figure 1:**
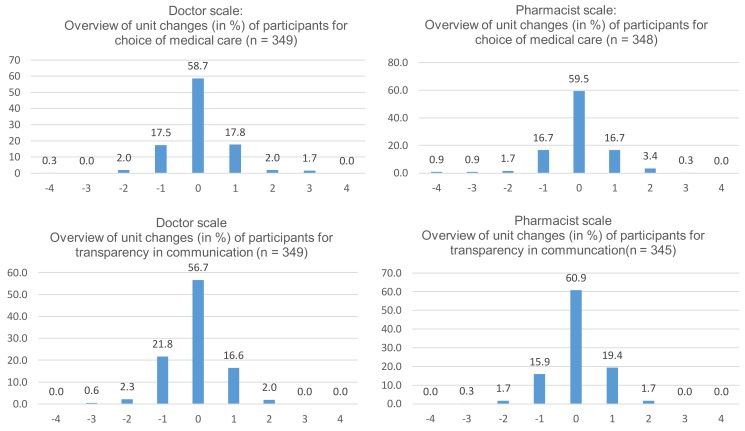

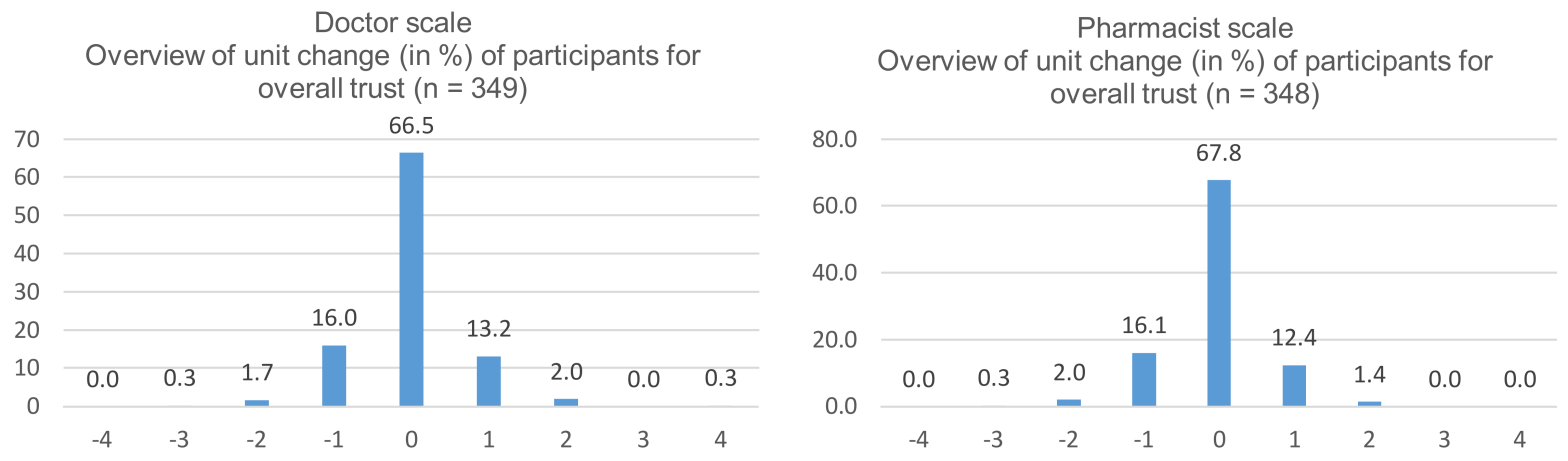
Individual unit change in level of trust.

Unit changes for doctor and pharmacist trust scales between baseline and 6-month for each question are presented in Figure 1, and illustrate a normal distribution of change in trust, indicating no shift in overall trust. In the vast majority of cases where trust either increased or decreased, it was a change in trust by only 1 unit on a 5-point scale. There were no significant associations between loss of trust and age, medication class, sex or intent to discuss deprescribing with either the doctor or pharmacist.

## 5. Discussion

From the patient’s point of view, receipt of a deprescribing intervention did not significantly alter pre-existing doctor or pharmacist trust. The majority of older adults either maintained or increased their level of trust in their healthcare provider after receiving a pharmacist-driven educational intervention to deprescribe inappropriate medications. Post-intervention changes in the level of trust followed a normal distribution, with 1-unit variations documented in either direction. Neither age, sex, intent to discuss deprescribing, nor type of medication targeted for deprescribing predicted loss of patient trust. Concerns remain unfounded that providing patients with education about medication harms will compromise the level of trust patients have in their doctor or pharmacist. 

This is the first study to investigate the association between patient-healthcare provider trust and deprescribing. The finding that patient and healthcare provider trust is not compromised by deprescribing is tangentially supported by the observation of Kerse et al. that high concordance between the patient and the doctor is associated with medication compliance [24]. Decisions about prescribing and deprescribing are likely not static, but may change over time, depending on a mutual understanding of the indications, benefits and risks of treatment. As deprescribing has been proposed as a patient-centered process [23], decisions that are taken in a personalized manner, depending on the needs and preferences of the individual patient, are more likely to promote trust than decisions that appear to be based on treatment guidelines that do not consider the individual’s perspective [25]. Bell et al. reported that doctors who believe that disclosing a medical error would diminish patient trust were more reluctant to disclose treatment errors [26]. Our results demonstrate that there was no overall shift in trust after the receipt of patient educational material about medication harms. Bell et al. also found that when doctors transparently disclose their notes to their patients, both the patient and the doctor gain relational benefits such as increased patient satisfaction and trust [26].

Pharmacists played a prominent role in promoting patient education in our study, serving as essential intermediaries between patients and physicians. Our results show that patients’ trust in their pharmacist is preserved in the context of a deprescribing intervention. Pharmacists and physicians identify similar classes of medications for deprescribing [27], highlighting the important role that community pharmacists can play as part of an interdisciplinary partnership with the family doctor. Preliminary results of the D-PRESCRIBE trial have demonstrated the incremental effectiveness in involving community pharmacists in the deprescribing conversation over and above direct-to-consumer education alone [28].

### Strengths and Limitations

This study used a validated questionnaire with excellent measurement properties. A large sample of community-dwelling older adults was surveyed with very few incomplete questionnaires, increasing external generalizability. There were no associations between loss of trust and age, sex, intent to discuss deprescribing, or medication class, indicating that education is generally widely acceptable to all patients. To preserve trial integrity, it was not possible to assess and compare temporal changes in trust between the control group and the intervention group. Likewise, participants were not asked to specifically state if the change was a result of the intervention. Therefore, it is impossible to determine if external factors were responsible for changes in trust. Although the PCAS has previously been validated, it was not possible to identify the test-retest variability over time. The normal distribution of the curves (Figure 1) may therefore be a representation of the normal variability of the survey, biasing the results towards no change, or regression to the mean.

## 6. Conclusions

As rates of polypharmacy rise among older adults, the need for deprescribing becomes imperative as an integral part of safe and appropriate healthcare for people aged 65 years and older. The results of this study show that patient-focused educational interventions to facilitate deprescribing result in no overall shift of patient trust in their healthcare provider. Therefore, prescribers can confidently provide patient-educational material as part of a strategy to improve deprescribing.

## Figures and Tables

**Table 1:** Targeted questions about trust from the Primary Care Assessment Survey.

Primary Care Assessment Survey Items	Likert Scale Response Options
I completely trust my doctor/pharmacist’s judgment about my medical care.	1—Strongly agree; 2—Agree; 3—Uncertain; 4—Disagree; 5—Strongly disagree
If a mistake was made in my treatment, my doctor/pharmacist would try to hide it from me.	1—Strongly agree; 2—Agree; 3—Uncertain; 4—Disagree; 5—Strongly disagree
All things considered, how much do you trust your doctor/pharmacist?	1—Strongly agree; 2—Agree; 3—Uncertain; 4—Disagree; 5—Strongly disagree

**Table 2:** Participant characteristics at baseline.

	**Patient Trust in Doctor**		**Patient Trust in Pharmacist**		**Total**
	Intact (*n* = 286) *n* (%)	Compromised (*n* = 63) *n* (%)	Intact (*n* = 284) *n* (%)	Compromised (*n* = 64) *n* (%)	(*n* = 352) *n* (%)
All participants					
Sex (female)	186 (82.3)	40 (17.7)	183 (81.0)	43 (19.0)	226 (64.8)
Age in years, Mean ± SD (range)	74.1 ± 6.4 (66–96)	75.5 ± 6.4 (66–92)	74.4 ± 6.6 (66–96)	74.1 ± 5.7 (66–90)	74.3 ± 6.4 (66–96)
Intent to discuss deprescribing	179 (82.5)	38 (17.5)	176 (81.5)	40 (18.5)	216 (62.6)
Participants by Medication Class:					
Benzodiazepine	166 (81.8)	37 (18.2)	164 (81.2)	38 (18.8)	203 (58.2)
First generation antihistamine	11 (100)	0 (0.0)	11 (100)	0 (0.0)	11 (3.2)
Long acting sulfonylurea	68 (76.4)	21 (23.6)	69 (77.5)	20 (22.5)	89 (25.5)
NSAID	41 (89.1)	5 (10.9)	40 (87.0)	6 (13.0)	46 (13.2)

SD = Standard Deviation; NSAID = Non-steroidal anti-inflammatory drug.

**Table 3:** Baseline and 6-month follow up scores on the trust questionnaire

Questionnaire Item	Doctors		Pharmacists	
	Baseline *n* (%)	Follow up *n* (%)	Baseline *n* (%)	Follow up *n* (%)
Trust in medical care *	*n* = 352	*n* = 349	*n* = 351	*n* = 349
Strongly agree	122 (34.7)	113 (32.1)	86 (24.4)	83 (23.6)
Agree	183 (52.0)	193 (54.8)	216 (61.4)	214 (60.8)
Uncertain	31 (8.8)	21 (6.0)	24 (6.8)	34 (9.7)
Disagree	13 (3.7)	15 (4.3)	16 (4.5)	15 (4.3)
Strongly disagree	3 (0.9)	7 (2.0)	9 (2.6)	3 (0.9)
Transparency in communication ^#^	*n* = 352	*n* = 349	*n* = 349	*n* = 348
Strongly agree	1 (0.3)	1 (0.3)	1 (0.3)	2 (0.6)
Agree	8 (2.3)	12 (3.4)	5 (1.4)	4 (1.1)
Uncertain	49 (13.9)	49 (13.9)	58 (16.5)	41 (11.6)
Disagree	208 (59.1)	219 (62.2)	225 (63.9)	247 (70.2)
Strongly disagree	86 (24.4)	68 (19.3)	60 (17.0)	54 (15.3)
Overall Trust ^~^	*n* = 351	*n* = 350	*n* = 351	*n* = 349
Not at all	3 (0.9)	3 (0.9)	2 (0.6)	3 (0.9)
Not Really	3 (0.9)	6 (1.7)	0 (0.0)	2 (0.6)
Somewhat	18 (5.1)	17 (4.8)	22 (6.3)	23 (6.5)
Mostly	109 (31.0)	110 (31.3)	157 (44.6)	163 (46.3)
Completely	218 (61.9)	214 (60.8)	170 (48.3)	158 (44.9)

* “I completely trust my doctor’s/pharmacist’s judgments about my medical care”; ^#^ “If a mistake was made in my treatment, my doctor/pharmacist would try to hide it from me”; ^~^ “All things considered, how much do you trust your doctor/pharmacist?”

**Table 4:** Post-intervention change in level of trust.

	Trust Level in Doctor (%)		Trust Level in Pharmacist (%)	
Questionnaire item	Increased/No change	95% CI	Increased/No change	95% CI
Trust in choice of medical care	78.5	74.2–82.8	79.4	75.3–83.9
Trust in transparency of communication	75.4	70.7–79.8	82.0	78.0–86.1
Overall trust	81.9	77.9–86.0	81.6	77.5–85.7
